# MRI Background Parenchymal Enhancement Is Not Associated with Breast Cancer

**DOI:** 10.1371/journal.pone.0158573

**Published:** 2016-07-05

**Authors:** Barbara Bennani-Baiti, Matthias Dietzel, Pascal Andreas Baltzer

**Affiliations:** 1 Department of Pharmaceutical Chemistry, University of Vienna, Vienna, Austria; 2 Department of Biomedical Imaging and Image-guided Therapy, Vienna General Hospital (AKH), Medical University of Vienna, Vienna, Austria; 3 Department of Radiology, University of Erlangen-Nürnberg, Nürnberg, Germany; University of Chicago, UNITED STATES

## Abstract

**Background:**

Previously, a strong positive association between background parenchymal enhancement (BPE) at magnetic resonance imaging (MRI) and breast cancer was reported in high-risk populations. We sought to determine, whether this was also true for non-high-risk patients.

**Methods:**

540 consecutive patients underwent breast MRI for assessment of breast findings (BI-RADS 0–5, non-high-risk screening (no familial history of breast cancer, no known genetic mutation, no prior chest irradiation, or previous breast cancer diagnosis)) and subsequent histological work-up. For this IRB-approved study, BPE and fibroglandular tissue FGT were retrospectively assessed by two experienced radiologists according to the BI-RADS lexicon. Pearson correlation coefficients were calculated to explore associations between BPE, FGT, age and final diagnosis of breast cancer. Subsequently, multivariate logistic regression analysis, considering covariate colinearities, was performed, using final diagnosis as the target variable and BPE, FGT and age as covariates.

**Results:**

Age showed a moderate negative correlation with FGT (r = -0.43, p<0.001) and a weak negative correlation with BPE (r = -0.28, p<0.001). FGT and BPE correlated moderately (r = 0.35, p<0.001). Final diagnosis of breast cancer displayed very weak negative correlations with FGT (r = -0.09, p = 0.046) and BPE (r = -0.156, p<0.001) and weak positive correlation with age (r = 0.353, p<0.001). On multivariate logistic regression analysis, the only independent covariate for prediction of breast cancer was age (OR 1.032, p<0.001).

**Conclusions:**

Based on our data, neither BPE nor FGT independently correlate with breast cancer risk in non-high-risk patients at MRI. Our model retained only age as an independent risk factor for breast cancer in this setting.

## Introduction

Breast density has evolved to be a major factor in clinical risk assessment of breast tissue in mammography [1–5]. In contrast to mammography, dynamic contrast enhanced magnetic resonance imaging (DCE-MRI) allows to differentiate between hormonally responsive glandular and fibrous connective tissues. High background parenchymal enhancement (BPE) is considered to correspond to hormonally active glandular tissue while fibroglandular tissue (FGT) values reflect the relative presence of breast parenchymal compared to fatty tissue. The former is particularly sensitive to hormonal changes and is thus higher in the second part of the menstrual cycle in younger women. Kuhl *et al*. and Mueller-Schimpfle *et al*. described already in 1997 incidental contrast-enhancing foci in otherwise healthy premenopausal women and noted that the enhancement velocities and number of resolvable lesions were lowest in the second week of the menstrual cycle [6, 7]. BPE is consequently also higher in younger women, in lactating women, and in women undergoing hormonal replacement therapy [8–11]. FGT has been linked to mammography density findings and was reported not to depend on contrast agent administration [12]. BPE is on the other hand largely independent from mammography density values [13–15]. Both BPE and FGT also vary considerably between patients and are currently reported using the American College of Radiology Breast Imaging Reporting and Data System (BI-RADS) [16–18].

Recently, two case-control studies described significantly increased odds of breast cancer in high risk patients with marked background enhancement [16,19]. However, it was also shown that breast tissues of women with BRCA1 and/or BRCA2 mutations are fundamentally different from those of women devoid of these genetic alterations, and this difference can be unveiled by MRI. Based on MRI findings of two-dimensional (2D) correlated spectroscopy (COSY), breast tissues featuring BRCA1/2 mutations exhibit significant changes in the lipid metabolism, including an increase of triglyceride and lipid unsaturation values in BRCA1 patients and cholesterol deregulation in BRCA2 patients [20]. It is thus plausible that BRCA1/2 mutations trigger a different metabolic make-up that renders findings from BRCA1/2 patients not readily transposable to patients lacking these mutations. We, therefore, sought to examine in a cross-sectional study whether enhanced background parenchymal enhancement constitutes a risk factor for breast cancer also in non-high risk patients. These were defined as women that were referred to MRI for diagnostic reasons that did not include patients deemed at high risk for breast cancer development as detailed in [21].

## Materials and Methods

### Patient selection

For this cross-sectional single-center IRB-approved retrospective study all 3020 patients that were referred to our department for breast MRI evaluation during a period of 44 months were eligible. IRB-approval was obtained from the Jena University Hospital Institutional Review Board ethics committee that waived the need for patient consent. Indications for breast MRI were further evaluation of suspicious conventional imaging findings, of discrepant findings between ultrasound and mammography, of clinical findings without imaging correlate (e.g. bloody nipple discharge) and follow-up of lesions classified as probably benign (corresponding to BI-RADS 0, 3, 4, 5 at conventional imaging). According to our institutional policy, any hormonal replacement therapy had to be ceased at least 6–8 weeks prior to examination. Patients had to have a reference standard established by histopathology to be included in this study. In case of benign findings, an additional imaging follow-up after a minimum of 12 months had to be documented. Consequently, 540 patients met our inclusion criteria. Median patient age was 54 years (mean age: 55 years (+/- 12.6, range 20–83). 60 patients were ≤ 40 years old, 220 were between 41 and 55 years old, and 260 patients were older than 55 years of age. Indications for breast MRI were distributed as follows: suspicious conventional findings BI-RADS ≥4 in 263 (111 already received a percutaneous 14G core biopsy before MRI), unclear or equivocal findings in 197, clinical findings only in 35 and follow-up of MRI detected BI-RADS 3 lesions in 20 patients. In 25 patients, the indication for the examination could not be retrieved from the institutional database.

### MR Imaging

All imaging was performed on 1.5 Tesla units (Magnetom Symphony and Magnetom Sonata, Siemens Medical Solutions, Erlangen, Germany). Dedicated vendor-supplied four-channel bilateral breast coils were used. The MRI-protocol adhered to international recommendations [22,23] and employed a dynamic sequence with 1-minute temporal resolution performed once before and seven times after automated injection (3 ml per second, Spectris, Medrad, Pittsburgh, USA) of 0.1 mmol/kg Gd-DTPA (Magnevist, Bayer Health Care, Leverkusen, Germany) into a cubital vein. Axial views of patients in prone positions were obtained. Subtractions were calculated by subtracting precontrast from postcontrast sequences.

### Reference Standard

Histopathological diagnosis was obtained after percutaneous image-guided biopsy for all lesions followed by subsequent surgery in case of malignant diagnosis. According to our institutions SOPs, all biopsy proven benign lesions were additionally followed-up by mammography and/or ultrasound for at least 12 months. This procedure was followed even if the benign lesion was surgically removed upon either patient or surgeon’s request. Board-certified breast pathologists with experience of more than 20 years in the field determined histopathological results.

### Data analysis

All MR-images were analyzed in chronological order by two experienced radiologists (> 500 Breast MRI examinations/year with more than twenty (WAK) and more than five years (PB) of dynamic contrast enhanced breast MRI experience) in consensus. Both were blinded to the results of the reference standard. The following parameters were visually determined for this study: background parenchymal enhancement (BPE; minimal/mild, moderate, marked); amount of fibroglandular tissue (FGT, a (1–25%), b (26–50%), c (51–75), d (76–100%). BPE was assessed on early enhanced scans 2 minutes after contrast medium injection by scrolling through the whole stack of DICOM images. Maximum intensity projections were not used as also minor motion artifacts lead to masking effects on BPE.

### Statistical analysis

The association between BPE and FGT and age and final diagnosis was explored by univariate Spearman rank correlation coefficient calculation. Cross-tabulated categorical data were analyzed by Chi-square statistics and continuous data by means of t-testing or ANOVA applying additional Scheffe’s post hoc testing. Odds ratios for diagnosis of breast cancer were calculated by univariate and multivariate binary logistic regression analysis. P-values below 0.01 were deemed to characterize significant findings for all calculations. The multivariate logistic regression analysis accounted for significant covariate collinearity by using interaction terms. All computations were performed using SPSS 22.0 (IBM, USA).

## Results

### Patient and lesion characteristics

540 patients were included in our study, exhibiting a malignancy rate of 65.4% (187 benign cases mean age of 51.76 years (median 52y, SD 12.5), 353 malignant cases mean age of 57.01 years (median 59y, SD 12.3). Age was lower in patients with benign lesions (p<0.001).

Since the menopausal status was not documented for the entire cohort, we stratified our patients into three different age groups. 60 patients were ≤ 40 years old (considered pre-menopausal), 220 individuals were between 41 and 55 years old (unclear menopausal status, likely peri-menopausal), and 260 patients were older than 55 years (considered post-menopausal).

Histopathology revealed 253 invasive ductal carcinomas (46.9%), 23 invasive lobular carcinomas (4.3%), 20 mixed invasive lobular-ductal carcinomas (3.7%), 24 other invasive carcinomas (4.4%) and 33 ductal carcinomas in situ (DCIS, 6.1%). The benign lesions were further classified into 63 cases of fibrocystic changes (11.7%), 22 fibroadenomas (4.1%), 34 papillomas (6.3%) and 68 other benign findings (i.e. inflammatory conditions, 12.6%). Among the malignant cases 74.2% tested positive for hormonal receptors.

### Association of BPE, FGT, age and cancer risk

**Fig 1:**
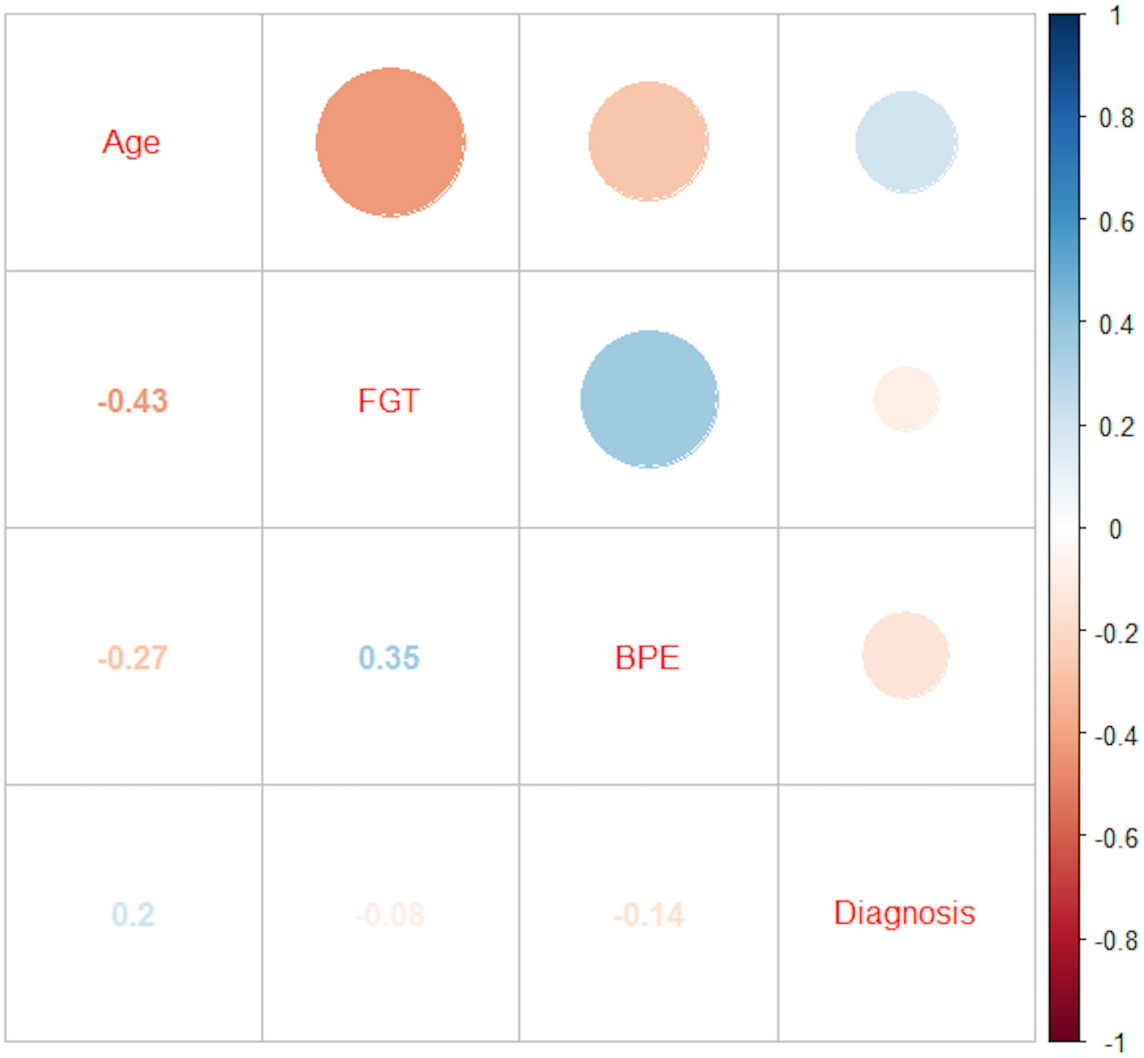
Color-coded correlation matrix of Spearman’s rank correlation coefficients. Blue indicates positive, whereas red indicates negative correlations. Size corresponds to strength of correlation. Correlation coefficient values are mirrored in the lower left quadrant. All values were statistically significant (p<0.001).

Table 1 depicts the distribution of malignant and benign cases according to age, BPE and FGT findings. Univariate testing identified a significant association between BPE and diagnostic outcome (χ^2^ 14.0, p = 0.001) but not for FGT and diagnostic outcome (χ^2^ 4.7, p = 0.195). Furthermore, malignancy rates differed between age groups (χ^2^ 17.7, p<0.001). Univariate Spearman rank correlation analysis unvealed highly significant negative moderate, negative weak and positive very weak correlations between age and FGT (rho -0.428), BPE (rho -0.279) and malignant diagnosis (rho 0.189), respectively (p<0.001). Both BPE and FGT displayed a very weak negative correlation with malignant diagnosis (rho^BPE:diagnostic outcome^ -0.156, p<0.001; rho^FGT:diagnostic outcome^ -0.086, p = 0.046). BPE exhibited a weak but statistically significant positive association with FGT (rho 0.353, p<0.001). These results are illustrated in Fig 1.

**Table 1:** Comparison of imaging characteristics between benign and malignant findings.

Characteristic	Benign cases	Malignant cases
BPE		
Minimal/mild	112 (29.6)	266 (70.4)
Moderate	48 (47.1)	54 (52.9)
Marked	27 (45)	33 (55)
FGT		
ACRa	29 (30.9)	65 (69.1)
ACRb	68 (30.9)	152 (69.1)
ACRc	65 (39.4)	100 (60.6)
ACRd	25 (41.0)	36 (59.0)
Age		
≤40 years	32 (53.3)	28 (46.7)
41–55 years	85 (38.6)	135 (61.4)
>55 years	70 (26.9)	190 (73.1)
Total	187 (34.6)	353 (65.4)

Data are numbers of subjects with percentages in parentheses, unless otherwise indicated.

### Odds ratios for presence of malignancy

Univariate analysis indicated that BPE was negatively associated with breast cancer risk (p = 0.001). Similarly, but statistically not significant, FGT was negatively associated with breast cancer risk (p = 0.054). On the other hand, age displayed a positive association with breast cancer risk (p<0.001). The multivariate analysis retained only age as an independent covariate associated with breast cancer risk (p<0.001).

**Fig 2:**
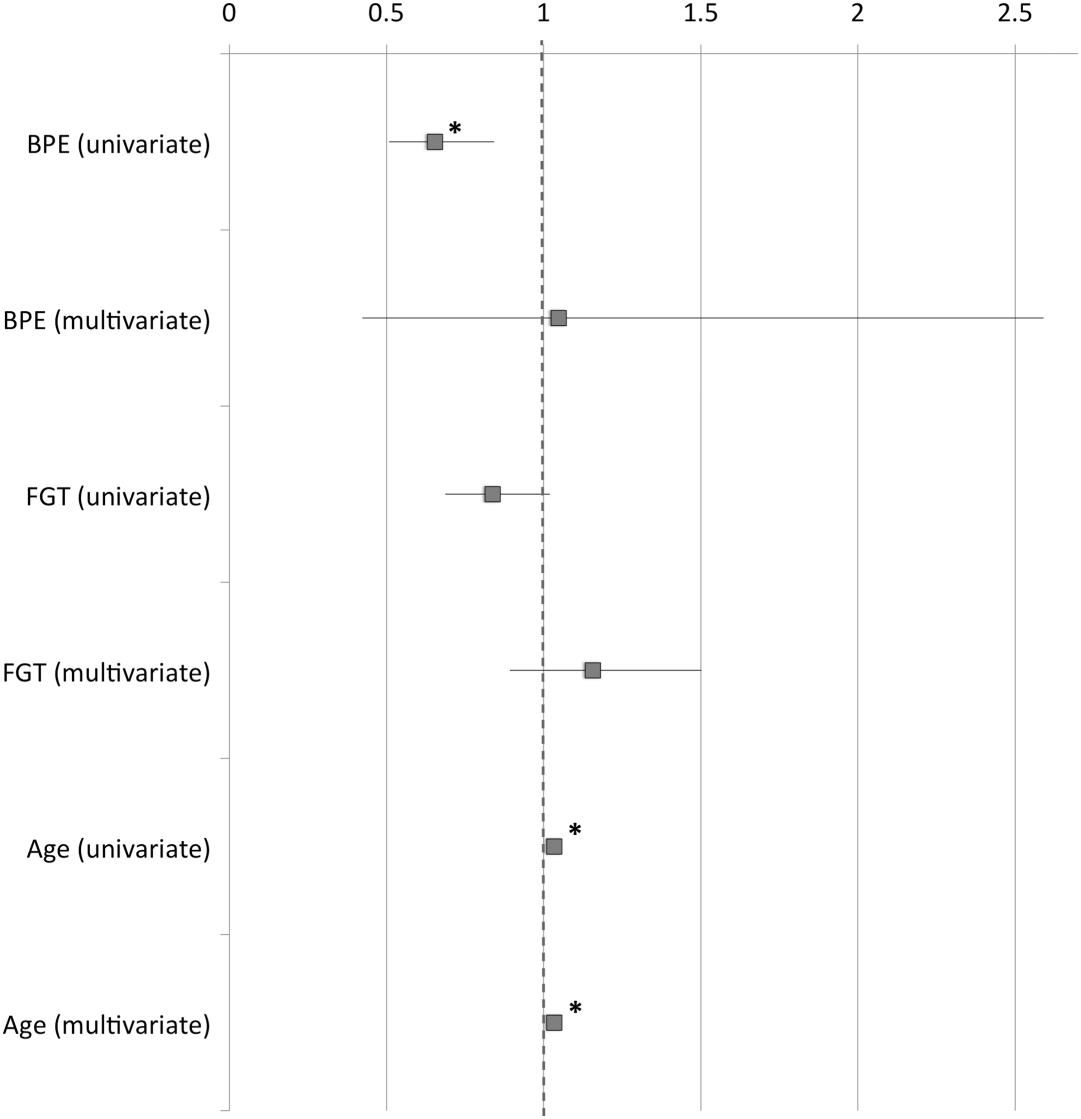
Odds ratios for presence of malignancy. Odds ratios for malignant diagnosis (squares) are indicated along with the respective 95%-confidence intervals (lines) for BPE, FGT and age as determined by multivariate and univariate analysis. * marks significant odds ratios in favor of (>1) or against (<1) malignant diagnosis (p< = 0.001).

Odds ratios and their 95% confidence intervals are given in Table 2 and Fig 2.

**Table 2:** Odds ratios for the investigated covariates regarding presence of breast cancer.

	Odds ratio (univariate)	p-value	Odds ratio (multivariate)	p-value
BPE	0.653 (0.507–0.841)	0.001	1.249 (0.469–3.332)	0.656
FGT	0.823 (0.675–1.003)	0.054	1.162 (0.895–1.508)	0.259
Age	1.034 (1.019–1.05)	<0.0001	1.032 (1.015–1.049)	<0.0001

95%-confidence intervals are given in parentheses.

### Association of Age with BPE

Patients with mild/minimal BPE (mean 57.5, median 59y, SD 12.3) were older than patients with moderate BPE (mean 50.8, median 50y, SD 12.2, p<0.001). Still younger were patients with marked BPE (48.5, median 47y, SD 11.3, p<0.001).

BPE differed significantly between age groups (χ^2^ 44.3, p<0.001). Patients ≤ 40 years were found to exhibit a higher proportion of moderate and marked BPE compared to patients between >55 years of age (χ^2^ 9.4, p = 0.002, Table 3).

**Table 3:** Cross-tabulation of age groups and BPE.

		BPE		
	minimal/mild	moderate	marked	Total
age				
≤40	28 (46.7)	18 (30)	14 (23.3)	60 (100)
41–55	136 (61.8)	50 (22.7)	34 (15.5)	220 (100)
>55	214 (82.3)	34 (13.1)	12 (4.6)	260 (100)
Total	378 (70)	102 (18.9)	60 (11.1)	540 (100)

Data are numbers of subjects with percentages in parentheses

### BPE and hormonal receptor status

The hormonal receptor status was available in a subset of 291 out of 353 malignant cases. Out of these, 74.2% were hormone receptor positive. The distribution of BPE categories and hormonal receptor status is given in Table 4, indicating that BPE and hormonal receptor status are not associated (χ^2^ 1.5, p = 0.475).

**Table 4:** Cross-tabulation of BPE and hormonal receptor status of malignant findings.

BPE	HR negative	HR positive
Minimal/mild	52 (24)	165 (76)
Moderate	14 (30.4)	32 (69.6)
Marked	9 (32.1)	19 (67.9)
Total	75 (25.8)	216 (74.2)

Data are numbers of subjects with percentages in parentheses

## Discussion

According to our results, BPE does not associate with breast cancer odds. The significant negative association between BPE and malignant diagnosis in univariate analyses owes to the high correlation of BPE with age. Age, however, maintained its positive correlation with breast cancer development consistently throughout our analysis. Put otherwise, BPE, which is a measure of tissue activity that decreases with age, is merely an indicator of age. Age, above all else, is the true prognostic factor, as uncovered by multivariate analysis.

To the best of our knowledge, these are the first data published on the correlation of BPE and breast cancer risk in non high-risk patients. There are, however, two recent studies by King *et al* and Dontchos *et al* in high-risk cohorts [16,19]. These two studies concluded that BPE correlated significantly with breast cancer risk and that elevated BPE resulted in as much as approximately 10-fold increased breast cancer odds. Both studies conceded that the sample size was rather small and too small to draw conclusions on specific subgroups especially relating to hormone-receptor status and BRCA1/2 status [16,19]. Unsurprisingly, these results implied to the community, that BPE was a risk factor for breast cancer independently of overall patient breast cancer risk. Yet, BPE, first and foremost, is a sensitive measure of tissue activity, one known to be driven by hormones and thus to closely correlate with age.

While, high density in mammography is an established breast cancer risk factor, no study has yet linked FGT to breast cancer risk. Neither Dontchos *et al*, nor King *et al* identified such a correlation, which is in line with our findings. This may also be due to the fact that our study was carried out in a non-screening setting, thus covering an entirely different patient cohort. Interestingly though, a recent editorial by Dolan *et al* stipulates that mammographic density does depend on individual patients´ risk [24].

**Fig 3:**
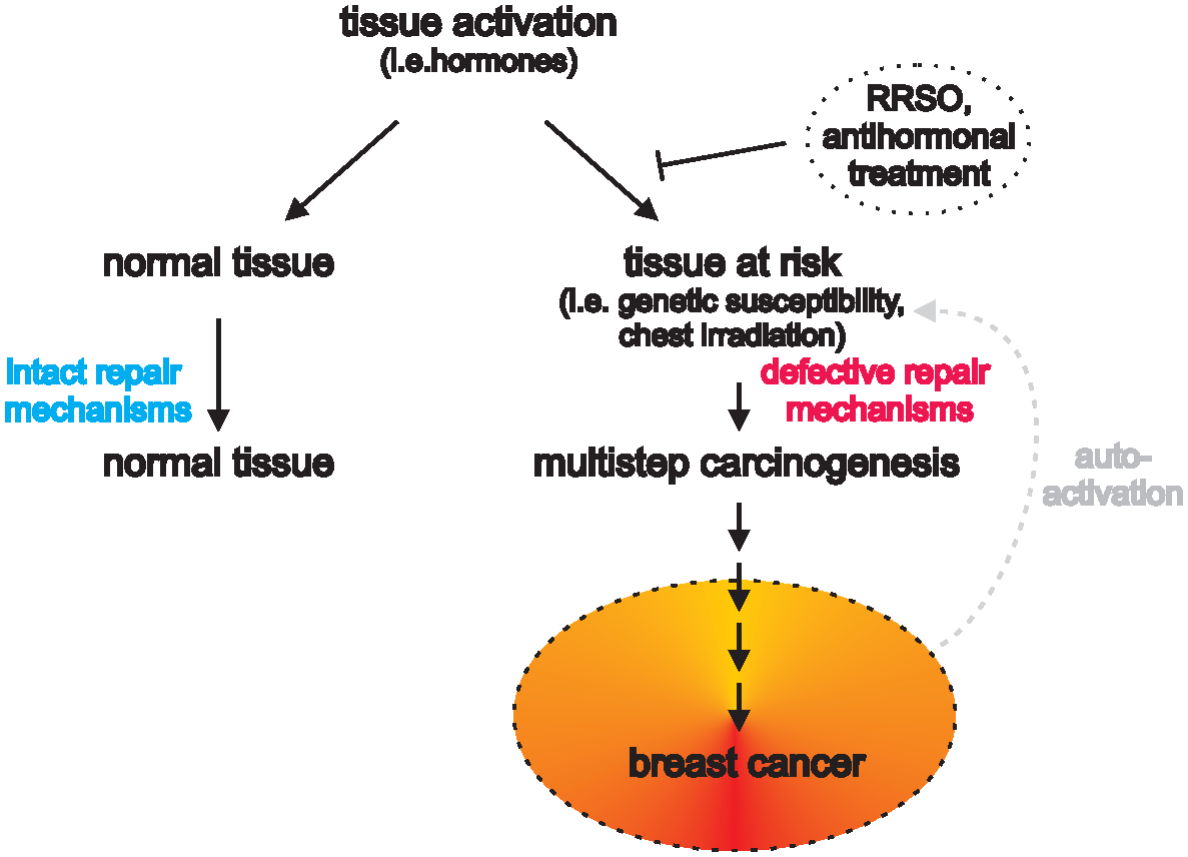
Tissue activation leads to breast cancer depending on tissue type. Hormones lead to breast tissue activation that is reflected by BPE levels in MRI. Normal tissues exhibit intact repair mechanisms (i.e. apoptosis, cell cycle arrest) that upon activation keep malignant transformation at bay. In breast tissues that lack these repair mechanisms (i.e. due to genetic defects or to genetic alterations due to radiation exposure), tissue activation will lead to a chain reaction that eventually causes malignant transformation. We speculate that tissue activation in this multi-step carcinogenesis becomes at one point no longer dependent on hormonal activation. Until then, reduction of endogenous hormones by RRSO or through antihormonal treatment can stop or slow down tissue activation as reflected by lowered BPE levels [27–30].

Given our data, the latter statement most probably also holds true for the correlation of BPE and breast cancer risk. Our findings, therefore, are in fact not in contrast with those of Dontchos *et al*. and of King *et al*., but rather are complementary to their observations. The crucial difference between our results and those of the aforementioned studies stems from the study populations considered. While the former investigated high-risk populations, we focused on non-high risk individuals. We specifically did not include patients deemed at high risk for breast cancer development due to an estimated life time risk of >20% based on individual (including prior breast or ovarian cancer and mantle field irradiation before the age of 30 years) and familial criteria or the presence of a genetic suscueptibility as detailed in [21]. As it were, breast tissue from high-risk patients is known to strongly differ from that of normal breast tissues. For instance, in BRCA1/2 carriers, BRCA1/2 mutations infer genetic instability, alter estrogen signaling, which ultimately causes significant histological remodeling; e.g. [25–27]. In patients whose tissue incurred genotoxic insults due to radiation or chemotherapy, other genetic mechanisms contribute to tissue vulnerability and may eventually lead to the formation of cancerous lesions upon cell growth stimuli or tissue activation. This can for example be observed following double-strand DNA damage commonly induced by radiation, which leads to genomic instability that can activate mechanisms of cellular escape from apoptosis and ensuing malignant growth; reviewed in [28]. Together, our data and the aforementioned studies provide evidence, at the imaging level, that tissue activity in tissue at risk (i.e. that harbors defects in its repair mechanisms) upon activation incurs sufficient alterations to progress towards malignant growth with time (Fig 3). As can be derived from the cross-tabulation of the correlation between menopausal status and BPE levels as well as the cross-tabulation correlating menopausal status to diagnostic outcome, post-menopausal age is associated with significantly lower BPE levels and significantly higher malignancy rates than pre-menopausal age. Further evidence for the hypothesis illustrated in Fig 3 is provided by three recent studies that demonstrated a strong correlation of BPE reduction after risk-reducing salpingo-oophorectomy (RRSO) in BRCA 1/2 carriers and subsequent cancer risk [18,29,30]. RRSO prompts a significant reduction of endogenous progesterone and estrogen levels, explaining the loss of tissue activation as indicated by BPE levels. Furthermore, van der Helden *et al*. reported a relatively poor outcome in patients with unilateral invasive estrogen receptor-positive, human epidermal growth factor-2 negative breast cancer, who underwent endocrine therapy only and exhibited low BPE values in the contralateral breast. In this instance, low levels of BPE indicated either low levels of or low tissue responsiveness to hormones to begin with, and therefore little possible gain from antihormonal therapy [31].

This all goes to say that BPE is only a measure of tissue activity and not a measure of breast cancer risk in non-high risk women. In instances wherein tissue activity is a contributing risk factor, BPE will correlate with breast cancer. It is important to note that women with normal breast tissue (i.e. no familial history of breast cancer, no known mutations, no previous chest irradiation) are not more susceptible to developing breast cancer when elevated levels of BPE are found at MRI. Given that BPE significantly correlates with age, and younger age equates to a lesser breast cancer risk, an elevated BPE measurement may even be regarded as a measure of healthy breast tissue. On the other hand, our data provide supporting evidence that BPE is a sensitive measure for tissue activity that can lead to breast cancer in tissues already prone to malignant transformation.

### Limitations of this study

Our work is not without limitations. Patients were recruited in a clinical, non-screening setting due to various conditions as listed in [22]. We employed histological verification as the reference standard for MRI findings. This biases the investigated sample towards a higher rate of malignancy. Contrary to previous studies [16,19], we chose a cross-sectional study design including consecutive patients referred to MRI. We addressed the confounders age and breast density by means of multivariate statistics while the case-control study design corrects for these variables by case matching. The latter is considered less favorable as it does not have the benefits of cross-sectional study design, including statistical power [32]. Our study design cannot however answer the question whether BPE is predictive of a breast cancer to develop in subsequent years. Here, a cross-sectional study with long-term follow-up would be needed.

For BPE and FGT assessment, we followed BI-RADS recommendations [17] and applied a qualitative visual assessment. Recent studies have probed more objective (semi-) automated approaches for quantifying BPE and FGT [33–36]. It is not unconceivable that such an approach could reveal associations between BPE, FGT and cancer odds that were too subtle for our visual approach. Finally, intraindividual variations of BPE according to the menstrual cycle phase have been observed in healthy volunteers. Consequently, it is recommended to schedule premopausal patients in the 2^nd^ week of their menstrual cycle. However, it is rare that patients show a significant increase in BPE during their menstrual cycle, e.g. from mild in the 2^nd^ week to marked outside of this period. Therefore, our institutional policy regularly schedules premoenopausal women for the 2^nd^ week of their menstrual cycle, unless highly suspicious findings at conventional imaging warrant immediate MRI evaluation. This practice could be seen as a potential confounder of our results. However, a prior retrospective analysis of our institutional data demonstrated only a minor difference in the prevalence of minimal/mild vs moderate/marked BPE comparing premenopausal women examined in the 2nd week of their menstrual cycle [37].

### Implications for patient care

Patients that are referred to MRI without previous breast cancer, known mutations, familial history of breast cancer or prior chest irradiation and found to exhibit elevated levels of BPE do not harbor an inherent increased risk of developing breast cancer. In these cases, elevated BPE is not an indication for more stringent follow-up diagnostic measures. If MRI reveals no additional malignant findings, the patient may return to routine screening.

## Supporting Information

S1 File SPSS table of the raw study data. (SAV)
