# Correction: MRI Background Parenchymal Enhancement Is Not Associated with Breast Cancer

**DOI:** 10.1371/journal.pone.0162936

**Published:** 2016-09-08

**Authors:** Barbara Bennani-Baiti, Matthias Dietzel, Pascal A. Baltzer

There is an error in the third author’s affiliation. Pascal A. Baltzer is not affiliated with #2 but is affiliated with #3: Department of Radiology, University of Erlangen-Nürnberg, Nürnberg, Germany

There is an error in the fourth paragraph of the Discussion section. The name “van der Helden” is incorrect. The correct name should be: van der Velden.

There is an error in the first sentence of the “Patient selection” subsection of the Material and Methods section. The correct sentence should read: For this cross-sectional single-center IRB-approved retrospective study all 3020 patients that were referred to the institute of diagnostic and interventional radiology at the university hospital Jena, Germany for breast MRI evaluation during a period of 44 months were eligible.
